# Endermic Use of Morphia by “Thimble Blistering”

**Published:** 1879-09

**Authors:** 


### ENDERMIC USE OF MORPHIA BY "THIMBLE BLISTERING."

Dr. Watson, in *Va. Medical Monthly*, describes a simple and sometimes very useful method of using morphia, whereby he claims that all danger is avoided and the annoying and sometimes even distressing symptoms which so frequently follow the use of opium, or its preparations, are escaped.

An ordinary sewing thimble, a little loosely picked up raw cotton, enough aqua ammonia (strong) to saturate cotton without running out, are the preliminary agents required. Gently press the thimble over the selected spot, until sensation of heat has been felt for 2 or 3 minutes—wipe away any ammonia which may remain on the surface—now with the finger rub away the superficial skin, apply dry morphia, by at first gently rubbing on, then carefully adding a drop of water. A small quantity may be repeated at short intervals until your patient feels its effects, or is satisfied with the relief obtained. The excess of morphia may then be wiped away. The author has used this method in about 100 cases with entire satisfaction.

